# Ethical Climate as Social Norm: Impact on Judgements and Behavioral Intentions in the Workplace

**DOI:** 10.3390/ijerph18116006

**Published:** 2021-06-03

**Authors:** Laurent Auzoult, Crisanta-Alina Mazilescu

**Affiliations:** 1 Psy-DREPI (Laboratoire de Psychologie: Dynamiques Relationnelles et Processus Identitaires), University of Burgundy, CEDEX, BP 27877-21078 Dijon, France; Laurent.Auzoult-Chagnault@u-bourgogne.fr; 2 Teacher Training Department, University Politehnica Timisoara (UPT), 300006 Timisoara, Romania

**Keywords:** ethical climate, social norm, regulation, judgmental and behavioral correlates

## Abstract

We present a study that looks at the relationship between the ethical climate, considered as a set of social norms, and judgments and behaviors in the workplace. In this case we think that an ethical rule reflecting the climate is only actualized in conduct and/or decisions if the rule is applicable, is shared, and is the subject of social expectations. A total of 277 professionals responded to a questionnaire measuring the normativity of three ethical rules, socio-moral judgment, trust in supervisors, turnover intention, and discrimination as well as abusive supervision. The results confirm our hypothesis. This leads to a different view of how the ethical climate is measured and constructed in the workplace.

## 1. Introduction

Exercising ethical and responsible management is a major challenge for most companies. Numerous studies [1,2,3,4] have shown that companies that adopt strategies based on ethics receive material and symbolic advantages in terms of profit and reputation.

Social psychology establishes that responsible social practices, i.e., the practice of producing goods and services in a way that is not harmful to society or the environment, depend on work activities, people, and organizational characteristics. Of these, the ethical climate seems to be an element that is fundamental for understanding the implementation of ethical social practices in the workplace.

We present a study on the consideration of the ethical climate as a social norm, i.e., shared expectations of acceptable behavior by group.

This study questions the way in which ethical behavior in the workplace, i.e., behaviour which respects the dignity, diversity, and rights of individuals and groups of people, must be perceived in order to give rise to effects on judgments and behavior in the workplace. Specifically, we seek to highlight the process by which ethical attitudes are actualized into judgments and behaviors consistent with responsible practices. This study proposes for the first time that it is when these attitudes become social norms that they are likely to translate into social practices.

This article begins with the presentation of research highlighting the impact of the ethical climate on organizational behavior. Then after clarifying the relationship between the social norm and behavior, we present a study that tests the relationship between the ethical climate measured as a social norm and a set of judgments and behaviors consisting of responsible practices.

## 2. Literature Review

### 2.1. Ethical Climate in the Workplace

Ethical climate refers to the shared perception of what is correct behavior, and how ethical situations should be handled in an organization [5]. The notion of an ethical climate accounts for a dimension of organizational functioning, which reflects employees’ perception of the rules of conduct, procedures, and practices related to professional ethics [6]. In this sense, the concept of an ethical climate refers to all the standards that serve as a regulatory framework for the behaviors expected in the organization.

Overall, the literature review by Newman, Round, Bhattacharya, and Roy [7] establishes that the origin of the ethical climate lies in organizational procedures and practices. Organizational culture and individual characteristics can also play a role in shaping the climate. The climate in turn impacts on psychological states, attitudes, intentions, and performance in the workplace [8,9].

For example, a high ethical climate is associated with positive attitudes at work but also with positive psychological health [10,11], performance [12,13,14], and high satisfaction [15,16] and a low turnover intention [17,18,19]. There is also a positive relationship between the ethical climate and commitment to work [20,21], trust in the organization [22], decrease corruption [23], and compliance with rules at work [24]. Finally, there is a relationship between the ethical climate and (un)ethical behavior at work or their antecedents [25,26,27,28,29,30,31] or decisions in the face of ethical dilemmas [32] or in the development of pro-environmental behaviors [33,34]. The impact of the ethical climate on behavior is based either on a mediating effect [14], or on a moderating effect. In this case the climate inhibits or promotes the impact of behavioral determinants [35] or judgment [36,37].

If the usual determinants and outcomes of the ethical climate are relatively circumscribed, there is little to help us understand the development and the impact of the ethical climate as a process. In their literature review, Ref. [7] indicate several theoretical pathways which highlight the impact of informational and normative social influences. In this sense, several authors envisage that the ethical climate may depend on the perception of behaviors and expectations coming from the social environment of employees [38]. As it is conceived, the ethical climate can therefore be considered as a set of norms which serve as a standard of regulation [6]. From a procedural point of view, it is therefore necessary to consider what leads a social norm to be updated in actual behavior.

### 2.2. From Social Norms to Behaviors

The studies referring to social norms [39,40] in particular to the impact of norms on behaviors [41], draw a distinction between subjective norms (the expectations of family and/or important people with regard to the behavior), injunctive norms (what others approve or disapprove of), descriptive and/or group norms (what others, relatives, and members of groups to which one belongs or people of personal significance usually do) and moral standards (what one personally considers is good or bad to do). Descriptive and injunctive norms reflect two facets of a single dimension of influence associated with the control and pressure of others [42] but one (the descriptive norm) refers to the regularity of behaviors or judgments within the group, and the other (the injunctive norm) to the punishable nature of deviance [43]. In addition, a distinction is made between the individual aspect (which conforms to personal principles) and the social aspect (what important people approve/disapprove of) of the injunctive norm [44].

Bicchieri [45,46] defines the conditions under which a social norm is updated in a behavior: A behavior rule (R) is a social norm for an individual (i) in a situation (S) insofar as:

a/the rule is known and recognized by i as applicable in S (contingency criterion);

b/it appears preferable to i to apply R provided that 2a. i believes that a large part of the population (P) applies R (empirical expectation criterion) and if 2b. i believes that P expects i themselves to apply R (normative expectation criterion) or 2c. i believes that P expects conformity with respect to R, expresses a preference for the conformity of i and appears ready to sanction the behavior of i (normative expectation with positive or negative sanction criterion).

To our knowledge, the model proposed by Bicchieri has only been tested empirically in a single study by Kubiszewski, Auzoult, Potard, and Lheureux [47]. In the latter, the authors were able to show that in the context of school bullying, the rules of behavior to be adopted towards victims, perceived as social norms, were associated with an intention to act.

### 2.3. Relation between Ethical Climate and Behavior

In their literature review, Wimbush and Shepard [30] underline that if the ethical climate influences ethical and even counterproductive behaviors, this relationship is conditioned by the relationship to others in organization. It is whether or not a decision on an ethical dilemma brings into play the interest or the consequences for oneself versus for others that determines the type of behavior that can take place. This finding is empirically confirmed [31] but is not taken into account in the way we theoretically consider the ethical climate nor in the way it is measured in studies predicting organizational behavior. In this study, we will test the hypothesis that behaviors within the ethical climate are associated with ethically driven judgments and behavioral intentions if, and only if, they are considered as social norms, i.e., that they meet the criteria defined by Bicchieri.

We have considered five correlates supposedly associated with the ethical climate: turnover intention, which refers to the intentions associated with mobility or staying in the organization; abusive supervision, which accounts for anti-social behavior at work on the part of line managers; trust in superiors, which indicates the belief that the relationship with the superior will be beneficial for their subordinate; socio-moral judgment, which accounts for the judgment of the perceived gravity of transgressive or atypical behaviors; and the propensity to discriminate, which refers to the intentions associated with discriminatory behavior in the workplace.

Specifically, we expected that the more ethical behaviors were perceived as social norms and the greater the intention to stay in the organization (*H*_1_), the higher would be the level of trust in supervisors (*H*_2_), and that of socio-moral judgment (*H*_3_). At the same time, we expected an inverse relationship between the normativity of ethical behaviors and turnover intentions (*H*_4_), the degree of abusive supervision (*H*_5_), and the propensity to discriminate (*H*_6_).

## 3. Materials and Methods

### 3.1. Participants and Procedure

The study was conducted in France by 277 volunteers (*n* = 238 females and *n* = 39 males, *M*age = 34.5 years). The participants worked in different professional sectors (health (22%)/social (38%)/trade (16%)/service, industry, civil service, and transportation (34%)). The sample size was not calculated a priori, as no study could be used as a basis for calculation. Data collection stopped after two reminders with potential participants. There is an imbalance in the gender of respondents.

The questionnaire that allowed us to measure the study variables was submitted via a professional forum dedicated to the publication of job offers. The message was disseminated by the Laboratory of the research team. Its contents indicated that the researchers were looking for volunteers to participate in study on workplace relationships. The inclusion criteria were to be active employees between 18 and 62 years old (legal retirement age), to work in organizations with more than 25 employees and to endorse the objective and participation in this study. Participants were invited to complete an online questionnaire and theirs answers were anonymous. Once data were completed and results processed, respondents received a report of the study’s main results by email (for participants who indicated an email).

### 3.2. Dependent Variables

**Normativity of ethical behaviors**: We used three behaviors present in the scale of Yener, Yaldiran, and Ergun [21]. The items referred to social responsibility (Rule 1; The people who work in my organization are actively concerned with the interests of customers and the public); professional rules and codes (Rule 2; In this company, employees are expected to strictly follow professional and legal standards); and personal interest and morals (Rule 3; In this business, what each employee thinks right or wrong is considered important). For the three behaviors, the participants had to indicate if the behavior could take place in their organization (cf. contingency), if it seemed desirable to them to adopt this behavior because it corresponded to what most employees do and think (cf. empirical expectation), and if it seemed desirable to adopt this behavior because it was what their colleagues expected/because it was valued by their colleagues/because people would not understand if they did not comply with it (cf. normative expectation). To code the normativity score, we considered a score of 1 for the standard (“Yes” response to the five items) and 0 for the lack of standard (at least one “no” response to one of the five items). The coding procedure uses the method of the study by Kubiszewski, Auzoult, Potard, and Lheureux, [47]. An affirmative response corresponded to the participant’s agreement respectively to: contingency criterion (This is a behavior that you feel can be implemented in your organization); empirical expectation criterion (You think it is desirable to adopt this behavior because it corresponds to what most employees do and think); normative expectation criterion (It seems desirable to you to adopt this behavior because it is what your colleagues expect from you; normative expectation with positive sanction (It seems to you desirable to adopt this behavior because it is valued by your colleagues) ; and normative expectation with negative sanction (It seems desirable to you to adopt this behavior because it would not be understood if you did not comply).

**Turnover intention**: we measured the different intentions using four items [48], namely, **the intention to quit externally** the organization (I intend to leave my job for another job elsewhere, in another organization; I will leave this job soon, no matter what happens), **the intention to progress internally** within the organization (I intend to leave my job for another job in my organization), and the **intention to rest** (I plan to stay in my job for many years). Participants responded using the five-point scale ranging from 1 “Not at all” to 5 “Completely”. The score was averaged after checking for internal consistency (Cronbach ‘alpha = 0.81).

**Abusive supervision**: we measured abusive supervision using 14 items [49]. Participants were asked to indicate whether their manager engaged in a number of anti-social behaviors (ridiculing, rudeness, lying, etc.), using a 5-point scale ranging from 1 (never behaves like this) to 5 (can sometimes or often behave like this). The score was averaged after checking for internal consistency (Cronbach ‘alpha = 0.94).

**Trust in the supervisor**: Confidence was measured using seven items [50]. The items referred to the behavior of the participants’ supervisor (I believe that my supervisor is a person of integrity; I can expect my supervisor to treat me in a consistent and predictable manner; My supervisor is not always honest and frank; Generally speaking, I believe that my supervisor’s intentions and reasons for acting are good; I do not believe that my supervisor is fair with me; My supervisor is open and frank with me; I am not sure that I can fully trust my supervisor). They responded using 5-point scales ranging from “Strongly disagree” to “Strongly agree”. The score was averaged after checking for internal consistency (Cronbach ‘alpha = 0.91).

**Socio-moral Judgment** in a professional environment: To measure socio-moral judgment we used the scale of [51] which includes 27 items. This scale measures the judgment of gravity in relation to moral transgressions (e.g., Pierre recently returned his service vehicle to his business park without reporting that he had hit a curb, seriously damaging the passenger door), transgression conventions (e.g., Marc sometimes comes to work in shorts despite the request of his superiors that he wear a uniform), and atypical behavior (e.g., at work, Timothy dresses as if he were permanently on holiday). The participants indicated their judgment of gravity vis-à-vis the different conducts using a 5-point scale. The score was averaged after checking for internal consistency (Cronbach ‘alpha = 0.78).

**Propensity to discriminate**: We measured the propensity to discriminate with the [52] scale. This scale offers three scenarios: A colleague asks you to sort CVs for the recruitment of a new team member by asking you not to select North African profiles (can be replaced by any target) (scenario 1); A colleague asks you to find them job seekers for a security post, specifying that they will only accept people of color (scenario 2); A colleague asks you to find them job seekers for a female cleaning post, during closed hours, saying that they will not accept women wearing headscarves (scenario 3). For these three scenarios, participants must indicate what behavior they would adopt: You carry out your colleague’s request, without talking to anyone about it, because you understand your colleague’s point of view; You execute your colleague’s request, without telling anyone, because you are afraid of breaking off your relationship with this colleague; You ask your interlocutor to make their request in writing so you can denounce the illegality of their request to your organization; You take the liberty of telling your interlocutor that this type of request is surprising and that you refuse to apply it. The answers correspond in descending order (from 4 to 1) to the intention to discriminate at work. The score was averaged after checking for internal consistency (Cronbach ‘alpha = 0.76).

## 4. Results

### 4.1. Common Method Variance

In order to control the Common Method Biases [53], we performed the *Harman’s single-factor test.* This test consists in performing an exploratory factorial analysis without rotation on all the items measured in the study. In this context, the fact of noting the emergence of a one-factor solution accounting for a high level of covariance led to the finding of a common variance bias. The analysis highlighted four factors with eigenvalues greater than one. The first factor accounted for only 28.45% of the variance for 66.87% for the four factors. This leads us to consider the risk of common variance bias as negligible.

### 4.2. Descriptive Statistics

The descriptive statistics are presented in Table 1. We corrected the correlations by attenuation [54] (Osborne, 2003). If we consider the norm globally by averaging the three normative behavior scores (*alpha* = 0.65), we see that the general normative score for the ethical climate is positively associated with the intention to stay (*r* = 0.24, *p* = 0.006, *H*_1_ verified), to trust (*r* = 0.28, *p* = 0.001, *H*_2_ verified), and with socio-moral judgment (*r* = 0.23, *p* = 0.01, *H*_3_ verified). Similarly, the general normativity score for the ethical climate is negatively associated with the level of abusive supervision (*r* = −0.26, *p* = 0.001, *H*_5_ verified) and with the propensity to discriminate (*r* = −0.21, *p* = 0.01, *H*_6_ verified).

More specifically, the normativity score of the first behavior (i.e., social responsibility) is positively associated with the intention to stay and with the level of trust in the supervisor and negatively with the intention to leave or progress internally within the organization and with the level of abusive supervision. The normativity score for the second behavior (i.e., professional rules and codes) is positively associated with the level of trust in the supervisor and socio-moral judgment and negatively with the level of abusive supervision. Finally, the normative score for the third behavior (i.e., personal interest and morality) is positively associated with the intention to stay, with the level of trust in the supervisor and socio-moral judgment and negatively with the level of abusive supervision and with the intention to discriminate.

Finally, after having averaged the socio-moral judgment scores on the moral (*alpha* = 0.57), conventional (*alpha* = 0.61), and atypical behavior (*alpha* = 0.70) registers, we find that the normativity of ethical behaviors is positively associated with conventional judgment (*r* = 0.16, *p* = 0.009), and atypical judgment (*r* = 0.14, *p* = 0.02) but is not associated with moral judgment (*r* = −0.05, *p* = 0.27). Regarding specific behaviors, hypothesis 4 is verified.

### 4.3. Relations between Norms and Correlates

We performed linear regression analyses by entering in dependent variables the set of judgment variables and in independent variables the three normativity scores for ethical behavior (Table 2). We observe that the normativity score of the first behavior explains the intention to progress internally and tends to the level of trust. The normativity score of the second behavior is associated with no judgment score. Finally, the normativity score of the third behavior explains the intention to stay and tends to explain socio-moral judgment and the intention to discriminate. Overall, even if relationships are few and relatively weak, the more normative are the ethical behaviors, the more the respondents’ judgments reflect a desire to remain in the organization, while maintaining relationships of trust, by issuing harsh judgments on transgressions and avoiding discriminatory behavior at work.

## 5. Discussion

In this study we are interested in the link between ethical behaviors within the ethical climate and a certain number of behavioral judgments and determinants at work. Unlike conventional work on climate, we have considered that climate should be perceived as a social norm in order for it to be reflected in judgments and behaviors of people in the workplace. We have referred to the model proposed by Bichierri to measure the normativity of ethical behavior, namely, on the assumption that these behaviors must be perceived as applicable, shared, and as being the subject of expectations and reinforcements coming from the social environment of employees. All of our assumptions were verified. The more ethical behaviors were perceived as social norms, the more they were associated with the intention to stay in the company via the adoption of harsh judgments of transgressions or the building of relationships based on trust with people in official positions of power. At the same time, the normativity of ethical behavior led to less potential discrimination and malevolent supervision. The climate measured as a social norm is positively associated with the intention to rest, thrust in supervisor, sociomoral judgment, and negatively to propensity to discriminate.

These results lead to two observations. On the one hand, it confirms the conditions defined by Bichierry [45,46] for defining and measuring a social norm as well as the results of Kubiszewski, Auzoult, Potard, and Lheureux [47]. It is by simultaneously taking into account the descriptive, subjective, and injunctive dimensions of the norms that we can predict the actualization of behaviors. This does not mean that the different dimensions taken in isolation cannot in certain cases predict behaviors. Indeed, in certain cases, for example, when the identity and the group are salient or when certain people exert a significant influence, the fact of sharing a behavior or knowing the expectations can be enough for this behavior to be adopted. At the same time, Bicchierri’s analysis leads us to think that when all the dimensions are effective, the prediction of behaviors is reinforced.

Furthermore, the fact that the normativity of behaviors is an important element in predicting judgments and ethical behaviors reinforces the idea that the adequate level for climate analysis, conceived as a set of ethical social norms, is not only the individual level of employees but that of the collectives making up the organization [7]. Measuring the social normativity of behaviors is an indirect measure of what can be represented at the collective level. However, it remains for future studies to confirm this hypothesis by simultaneously taking into account several levels of analysis and measurements.

This study is the first to consider the ethical climate as a construct to be taken into account theoretically and empirically as a social norm. From this point of view, it should be specified in future studies how this type of standards can be implemented in organizations and how they can interact with other organizational standards.

This study is based on a cross-sectional study and in this sense does not lead to unequivocal conclusions on the link between normativity of behavior and ethics and effective decisions at work. Nonetheless, it suggests that a main determinant of the construction of the ethical climate is *shared reflexivity*, namely, the ability of employees to talk about and discuss work ethics. This type of discussion would in fact be likely to support the descriptive and subjective nature of the normative behavior. We can also think that the leadership exercised is decisive in terms of conditions for the social modeling of behavior. Other studies would be relevant to take into account the limitations of this study. In particular, it would be appropriate to implement an experimental design that would confirm the causal link that this study suggests between climate normativity and judgments or organizational behavior. Another objective would be to take into account cultural and gender differences. Indeed, females showed significantly more favorable attitudes towards ethical behaviors than males [55]. From this perspective, the relationships we observed may be favored by the fact that our sample was biased from point of view of gender.

## 6. Conclusions

In this study, we demonstrated that by measuring the ethical climate as a social norm, i.e., as a set of rules perceived as applicable and expected by others, we could predict behaviors considered as ethical. This study proposes a new conception of the ethical climate. By considering the climate as a social norm, we offer a new theoretical perspective but also practical avenues. Indeed, the climate can sometimes appear as a construct that describes organizations but provides few regulatory levers for behavior. From this point of view, this study suggests that highlighting the applicability of rules of conduct and social expectations (of supervisors, the organization, and customers) can lead to limiting unethical behavior and promote ethical behavior. This practical potential remains to be confirmed in future studies.

## Figures and Tables

**Table 1:** Correlation matrix between all variables (Alpha de Cronbach between brakets).

Variables	M	*SD*	1	2	3	4	5	6	7	8	9	10
Rule 1µ_1_	1.46	*0.49*		0.40	0.41	−0.13 *	−0.14 *	0.14 *	−0.16 *	0.19 *	0.07	−0.10
Rule 2 µ_2_	1.51	*0.50*			0.33	−0.04	−0.06	0.07	−0.16 *	0.15 *	0.14 *	−0.09
Rule 3 µ_3_	1.61	*0.49*				−0.08	−0.02	0.17 *	−0.14 *	0.17*	0.15 *	−0.15 *
Intention to quit externally	2.58	*1.37*				(0.81)	0.17 *	−0.72 *	0.37 *	−0.40 *	−0.02	0.12 *
Intention to progress internally	2.20	*1.28*						−0.14 *	0.10	−0.15 *	0.08	0.04
Intention to rest	2.87	*1.46*							−0.41 *	0.44 *	0.04	−0.12 *
Abusive supervision	1.71	*0.78*							(0.94)	−0.76 *	0.02	−0.03
Thrust in supervisor	3.49	*1.03*								(0.91)	0.001	0.06
Sociomoral judgement	3.26	*0.35*									(0.78)	−0.07
Propensity to discriminate	3.70	*0.51*										(0.76)

Note: Rule 1 µ_1_ (The people who work in my organization are actively concerned with the interests of clients and the public), Rule 2 µ_2_ (In this company, employees are expected to strictly follow professional and legal standards), Rule 3 µ_3_ (In this business, what each employee thinks right or wrong is considered important); * *p* < 0.05.

**Table 2:** Regression analyzes—norms in independent variables and correlates of judgment in dependent variables.

	Intention to Quit Externally	Intention to Progress Internally	Intention to Rest	Abusive Supervision	Thrust in Supervisor	Sociomoral Judgement	Propensity to Discriminate
Norm 1	−0.12	−0.15 **	0.09	−0.09	0.13 *	−0.02	−0.04
Norm 2	0.02	0.01	−0.01	−0.10	0.06	0.10	−0.03
Norm 3	−0.03	0.05	0.14 **	−0.07	0.10	0.12 *	−0.12 *
R^2^	0.02	0.02	0.04 **	0.04 **	0.05 **	0.03 **	0.02

Note: * *p* < 0.10; ** *p* < 0.05.

## Data Availability

Data available on demand.
